# Nanomaterial Probes for Nuclear Imaging

**DOI:** 10.3390/nano12040582

**Published:** 2022-02-09

**Authors:** Vanessa Jing Xin Phua, Chang-Tong Yang, Bin Xia, Sean Xuexian Yan, Jiang Liu, Swee Eng Aw, Tao He, David Chee Eng Ng

**Affiliations:** 1 Department of Nuclear Medicine and Molecular Imaging, Radiological Sciences Division, Singapore General Hospital, Outram Road, Singapore 169608, Singapore; vanessa.phua.j.x@sgh.com.sg (V.J.X.P.); sean.yan.x.x@singhealth.com.sg (S.X.Y.); aw.swee.eng@singhealth.com.sg (S.E.A.); david.ng.c.e@singhealth.com.sg (D.C.E.N.); 2 Duke-NUS Medical School, 8 College Road, Singapore 169857, Singapore; 3 School of Chemistry and Chemical Engineering, Hefei University of Technology, Hefei 230009, China; xiabin@mail.hfut.edu.cn (B.X.); taohe@hfut.edu.cn (T.H.); 4 Department of Computer Science and Engineering, Southern University of Science and Technology, 1088 Xueyuan Avenue, Shenzhen 518055, China; liuj@sustech.edu.cn

**Keywords:** nanomaterials, nanoparticles, molecular imaging probe, nuclear imaging, theranostics

## Abstract

Nuclear imaging is a powerful non-invasive imaging technique that is rapidly developing in medical theranostics. Nuclear imaging requires radiolabeling isotopes for non-invasive imaging through the radioactive decay emission of the radionuclide. Nuclear imaging probes, commonly known as radiotracers, are radioisotope-labeled small molecules. Nanomaterials have shown potential as nuclear imaging probes for theranostic applications. By modifying the surface of nanomaterials, multifunctional radio-labeled nanomaterials can be obtained for in vivo biodistribution and targeting in initial animal imaging studies. Various surface modification strategies have been developed, and targeting moieties have been attached to the nanomaterials to render biocompatibility and enable specific targeting. Through integration of complementary imaging probes to a single nanoparticulate, multimodal molecular imaging can be performed as images with high sensitivity, resolution, and specificity. In this review, nanomaterial nuclear imaging probes including inorganic nanomaterials such as quantum dots (QDs), organic nanomaterials such as liposomes, and exosomes are summarized. These new developments in nanomaterials are expected to introduce a paradigm shift in nuclear imaging, thereby creating new opportunities for theranostic medical imaging tools.

## 1. Introduction

Molecular imaging is a non-invasive medical imaging technique capable of providing detailed images and information at molecular and cellular levels. Molecular imaging enables the visualization of cellular function through the different imaging modalities such as Positron Emission Tomography (PET) and Single-photon Emission Computed Tomography (SPECT). Imaging probes or biomarkers are used for tracking specific molecular pathways in particular targets in a living system [1,2]. The molecular imaging techniques offer possibilities of early detection and treatments of diseases through molecular imaging. Through a variety of novel molecular imaging applications, understanding of pathological development is expected to be enhanced, facilitating drug discovery and development in tackling diseases [3].

Bionanomaterials including organic and inorganic materials can be easily assimilated in living systems. These small-sized nanomaterials can penetrate into tiny capillaries and propagate across biological barriers, enabling detection of changes occurring at molecular levels. Combining these unique properties with being biocompatible has accelerated the application of nano-biomaterials for molecular imaging [4]. Nanomaterials can be applied as a probe for various imaging modalities such as PET/SPECT, Computed tomography (CT), Magnetic Resonance Imaging (MRI), Ultrasound (US), optical imaging, etc. An advantage unique to nanomaterials is their large surface area-to-volume ratio, which can carry not only a large “payload” of probes at the surface but also targeting moieties or ligands, leading to a favorable biodistribution pattern of the nanomaterials in living systems. Nanomaterial probes can target areas of inflammation or tumors through passive targeting by so-called enhanced permeation and retention (EPR) effects. The imaging technique used for enhanced permeation and retention is referred to as perfusion imaging. Typically, the imaging probe is injected and monitored continuously to assess how fast the material goes into affected tissue (wash-in) and cleared from the same affected tissue (wash-out). The perfusion imaging can be used to measure the vascularization in stroke, tumor, and even inflammation, for example, to assess the amount of reperfusion that occurs in the area of an infract post-treatment, to gauge the recovery of the patient from stroke [5], and to assess anti-angiogenic, radiation, and chemotherapeutic treatment responses in oncology [6].

Nuclear imaging PET and SPECT are the modality techniques with high sensitivity. The neutron-deficient or proton-rich radioisotope undergoes positron decay, resulting in further annihilation to produce two photons that travel in opposite direction with an energy of 511 KeV. These annihilation events are collected into sinograms through tomographic techniques [7]. The physics behind each molecular imaging technique determines the design of the nanomaterials. The nanomaterials are designed with biocompatibility for each diagnostic application. For example, the CT technique is useful for high-resolution anatomical imaging when incorporated with other tracer imaging modalities such as PET or SPECT. As the dose administered for PET and SPECT tracers are in the order of the nanomolar (nM) range, they are considered to have low toxicity and be biocompatible [8]. Currently, the most commonly used radionuclides in clinical practice are fluorine-18 (^18^F), carbon-11 (^11^C), gallium-68 (^68^Ga), iodine-124 (^124^I), and copper-64 (^64^Cu) for PET and technetium-99m (^99m^Tc) for SPECT. ^18^Fluorinated 2-deoxy glucose (^18^F-FDG) is one of the most extensively used PET tracers. The design of ^18^F-FDG is for detecting the higher expression of glucose transporters in cancer cells [9]. The 2-deoxyglucose (2-DG) is excessively taken up by the tumor as upregulated glucose transporters are phosphorylated in the glycolysis pathway, while the normal tissue has an insignificant uptake of 2-DG. Employment of ^18^F-FDG helps in the assessment and treatment of solid tumors using PET.

Although small molecule tracers such as FDG are the most widely used clinical PET radiotracers, there is a need to develop nanomaterial-based nuclear imaging probes, as the small molecule ones exhibit fast metabolism and non-specific distribution [10]. Nanomaterials or nanoparticles have been used as a platform to carry radiopharmaceuticals for specific targeting with other multi-functionalities. Multifunctional nanomaterials or nanoparticles have been of interest due to a number of advantages: surface modification such as coating for biocompatibility, favorable blood circulation, and easy to be manipulated for functional group attachment for enhanced targeting ability. Due to the size of nanoparticles, which are normally 100–10,000 times smaller than cells, nanoparticles can be easily tailored for cell internalization [11]. More excitingly, the wide loading ability of nanomaterials has made it possible to simultaneously load diagnostic and therapeutic moieties into one package for theranostic applications [12,13,14,15].

Commonly used non-invasive molecular imaging modalities include PET, SPECT, MRI, optical imaging, CT, etc. Each imaging modality has distinct advantages, while there are also inherent limitations, hindering a single imaging modality in providing all required information. Nuclear imaging techniques such as PET and SPECT are highly sensitive and provide deep penetration into tissues by using γ-ray emission; however, they are compromised by low spatial resolution [12,14]. On the other hand, imaging modalities such as MRI and CT provide high spatial resolution but have relatively low sensitivity. Hence, the combination of different imaging modalities is favorable as a diagnostic tool for providing detailed information. For example, combining the two modalities of PET and MRI offers complementary information such as deep tissue penetration and three-dimensional (3D) anatomical information. Bimodal PET/MRI imaging is already implemented and widely used in clinical practices. Development of multimodal imaging probes can help in the identification and positioning of abnormal tissues accurately via complementary physiological and anatomical information attained from PET or SPECT and MRI, respectively.

**Scheme 1:**
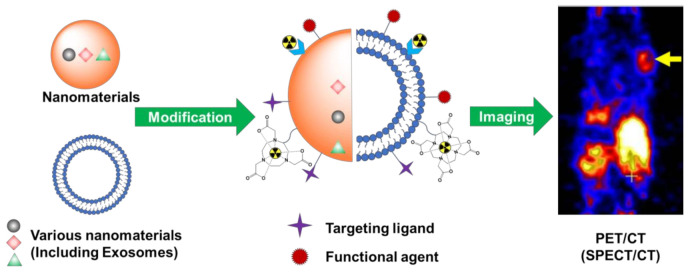
Various nanomaterials through modifications for nuclear imaging PET/CT (SPECT/CT) probes.

In this review, we discuss some key factors of nanomaterial modification for their applications as nuclear imaging probes in preclinical and potential clinical uses. We summarize the recent progress in the development of nanomaterials including organic nanomaterials such as peptides, antibodies, liposomes, and inorganic nanomaterials as nuclear imaging probes (Table 1). The development of exosomes as nuclear imaging probes is highlighted (Scheme 1). Most of the nanomaterial nuclear imaging probes selected and described in this review were reported in the past five years.

## 2. Challenges of Nuclear Imaging and the Role of Nanomaterials

A key challenge faced in current nuclear imaging is the design of an imaging probe that is suitable for clinical application. An ideal imaging probe includes but is not limited to the following properties: biocompatible, easily excreted from the body, enables robust imaging signal and sensitivity, site-specific targeting to area of interest, physiochemical behavior of probe in relation to radioisotope, low toxicity, and good biodistribution [1]. Additionally, the selection of a radioisotope for radiolabeling is also crucial in the design of the probe. There are certain criteria to consider in the development and design of an optimal probe. Sufficient half-life and optimal energy of the radioisotopes have to be complementary to the selected probes for quality imaging [44]. For example, ^68^Ga and ^18^F for PET are radioisotopes with a short half-life utilized for imaging purposes with drugs that have fast distribution kinetics. On the other hand, longer half-life radioisotopes such as ^64^Cu are commonly utilized with antibodies for site-specific targeting, which requires a period of time to reach. Small molecules such as FDG are currently used as imaging probes in PET imaging. However, small molecules tend to exhibit non-specific distribution and poor uptake in certain tumors, which could lead to deviation from diagnostic results [2,10,45]. Single biomolecules can only transport one radioisotope to targeted sites [46]. Large molecules and more than 98% of small molecules, imaging probes or drugs do not have the ability to propagate across the blood–brain barrier to target neurological or intracranial diseases [47].

Nanomaterials can potentially overcome these limitations due to their advantageous size and physiochemical properties [4]. The nanosized feature allows localization of probes at disease sites and reduction in renal excretion and metabolism in the liver due to the unique properties of EPR, enabling prolonged circulation in the body [46]. The pharmacokinetic behavior and biodistribution of the nanomaterial probes allow their theranostic applications with regard to various preclinical and clinical objectives. Effective radiotherapy requires an efficient amount of radiopharmaceutical payload to be delivered to the targeted sites (high payload). For a single nanoparticle, multiple radioisotopes can be labeled, thereby transporting hundreds of radionuclides to the targeted sites. The increase in therapeutic payloads makes nanomaterials effective radiotherapy probes. Moreover, the potential in integrating multiple complementary radioisotopes and signal reporters on a single nanoparticle allows the usage of multimodal molecular imaging. Additionally, quantitative imaging can be performed through the incorporation of radioisotopes onto nanoparticles using PET or SPECT for in vivo biodistribution studies, which are essential for the evaluation and validation of potential clinical therapeutics.

The limitations of crossing biological barriers such as the blood–brain barrier (BBB) and small capillaries can also be overcome using nanomaterials. These nanosized vessels are small enough to propagate across the barriers, delivering drugs to targeted sites [4]. For example, Dalargin, an antineoplastic drug, is unable to propagate across the BBB when administered systematically [48]. However, the conjugation of [^3^H]Dalargin to poly(butylcyanoacrylate) nanoparticles injected systematically showed accumulations in the brain, indicating that the nanoparticle complex had crossed the BBB. The development of nanomaterials for specific targeting of the Central Nervous System (CNS) still remains extremely challenging due to the safety and efficacy concerns. However, there has been great advancement in the design of nanoparticle as carriers, drugs or probes to efficiently and safely propagate across the BBB. With more knowledge and better understanding of nanomaterials and their mechanisms, more success in the design of a nanomaterial probe may be achieved in targeting neurological diseases in the near future, creating more opportunities in therapeutics and diagnostic clinical applications.

Bionanomaterials in particular have been widely investigated for theranostic applications due their natural ability to assimilate easily in body systems. These natural nanoparticles are equipped with unique properties such as biocompatibility, biodegradability, and stability in blood circulation, thereby overcoming some of the limitations that need to be considered in the design and development of a novel imaging probe [49]. For example, metallic bionanomaterials and their oxides such as gold, silver, copper, and iron are easily engineered and synthesized and have shown low toxicity for both living cells and the environment, making these a favorable drug or imaging probe to be used in diagnosis and therapeutic applications. Enhancement of molecular imaging can also be achieved as bionanomaterials not only provide biocompatibility but also have a high surface area per volume, resulting in a high payload and selective binding to targets via passive or active targeting that enables specific pathway signals to be elicited for detection using imaging modalities at molecular levels. With these unique features of bionanomaterials, more accurate diagnosis of diseases can be attained and treatments can be tailored accordingly.

## 3. Modifications of Nanomaterials for Nuclear Imaging

**Scheme 2:**
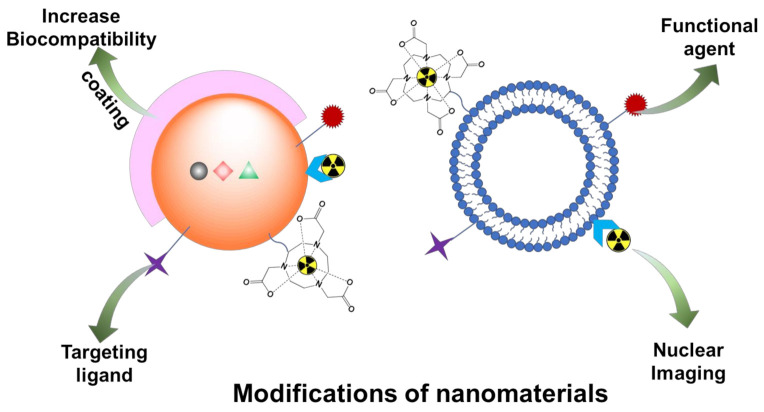
Various modifications of nanomaterials as nuclear imaging probes for their multi-functional theranostic applications.

To improve their biocompatibility, targeting specificity, and easily controllable properties, nanomaterial-based nuclear imaging probes require necessary surface modifications such as coating and targeting moiety attachments, which include conjugation with peptides, antibodies, and many other targeting molecules (Scheme 2). The modifications promote various advantageous properties such as site-specific targeting, higher affinity to biomolecules, enhanced uptake into cells, improved biocompatibility, and longer circulation time that are essential for theranostic applications.

### 3.1. Coating

The nanomaterial probe administered intravenously into the living system is assimilated either by the hepatobiliary system or by the renal system. To improve the biocompatibility, the nanoparticles are coated with hydrophilic polymers that include linear chain polymers of polyethylene glycol (PEG) and branched copolymers [50,51]. The hydrophilic nature of the coating polymer prevents ‘opsonisation’, as the hydroxyl (-OH) group offers steric hindrance to the approaching protein. It has been shown that the particles coated with PEG have a tendency to evade opsonisation by the immune system [52]. It was found that an increase in the molecular weight of PEG resulted in nanoparticles with longer blood circulation time but also resulted in reduced tumor accumulation [53,54,55]. Both the concentration and morphology of the PEG chains affect the targeting capability of the nanomaterial imaging probe. At a moderate concentration, PEG chains presented a brush conformation due to the lateral pressure between these PEG chains while the PEG chains would tangle themselves into a random-coil conformation known as the mushroom conformation when PEG density was too high [56]. The brush conformation PEG chains grafted on targeting moieties would have more chance to interact with receptors [57]. To achieve specific targeting capability, the concentrations and morphology of PEG chains need to be carefully optimized.

Apart from PEG, several other coating polymers including polyvinyl pyrrollidone (PVP) [58], polyvinyl alcohol (PVA) [59], polylactide, polycaprolactone, polaxomers of polyethylene oxide (PEO), and polypropylene oxide (PPO) have been developed. Cyclodextran, a natural polysaccharide with an inert property, is most commonly used for coating iron oxide nanoparticles (IONPs), enabling longer circulation in living systems [60,61,62]. The liposomes with amphiphilic long-chain lipid molecules form a lipid-bilayer around the signaling molecule and mimic the plasma membrane of the blood corpuscles, thereby overcoming the reticuloendothelial (RES) system and providing a long circulation time in the blood [63,64,65]. Apart from linear chain polymers, branched amphiphilic copolymers such as dendrimers show superior biocompatibility with improved blood circulation time. The smaller size (~20 nm) dendrimers show higher efficiency of ghost drug encapsulation [66].

Normally, nanomaterials are conjugated with functional linkers for further improved pharmacokinetics and biodistribution. Linkers bound with “targeting ligands” have high affinity. The most commonly used system is biotin–avidin. Biotin a water-soluble vitamin that can bind to streptavidin molecules with a very high affinity, defined by the dissociation constant K_D_. Dissociation constants determine the affinity with which the molecules can bind to each other. Melamide, succinimide, and other carbodiimide reactions are exploited to form covalent ester bond to link typically an alcohol group–carboxylic group to create an ester bond. Many such reactions are useful in molecular imaging for functionalizing the surface of the nanoparticles [67,68,69].

### 3.2. Active Targeting Moieties for Disease-Specific Receptors

Nanomaterial itself in which the radiolabeled isotopes are packed and delivered to the living system is essential for non-specific uptake and ideal distribution behavior for molecular imaging. The nano-sized particles offer a greater surface area per volume of the particle, giving accessibility for functionalization of the surface with ligands including biological peptides, antibodies, vitamins, and other biological molecules. These nanoparticles can selectively bind to cell surface receptors and cause specific “signal” changes that can be detected. The “signals” are due to the behavior of the nanoparticle. Targeting moieties have been designed to bind to receptors that are over-expressed in pathological cells but much less expressed in normal cells [70,71]. The targeting moieties differentiate pathological cells from healthy cells, actively enhancing the specific cell binding. The targeting moieties can facilitate the nanoparticle localization in lesions [72]. Targeting moieties are normally conjugated or loaded onto the outer surface of nanomaterials to achieve maximum receptor binding. Commonly used targeting moieties are peptides such as somatostatin and cyclic arginylglycylaspartic acid (cRGD), antibodies such as Traztuzumab, and small molecules. Somatostatin is a targeting poly-peptide as it binds to the over-expressed somatostatin receptors (SSTRs) presented on the neuroendocrine tumors. Its analog poly-peptides such as octreotide bind to SSTR with very high affinity, leading to a combined targeting and therapeutic effect. Cetuximab, an epidermal growth factor receptor inhibitor showed therapeutic effects in metastatic cancer [73]. Cyclic arginylglycylaspartic acid peptide (cRGD) is another small peptide that binds to α_v_β_3_ integrins, which are over-expressed during neovascularization in cancer. cRGD has been used as a molecular imaging target to evaluate neovascularization in tumors. cRGD conjugated superparamagnetic iron oxides (SPIOs) as MRI contrast agents showed excellent distribution in the tumor vasculature, which was not observed in control tumors. cRGD functionalized SPIO conjugated 1,4,7,10-tetraazacyclododecane-1,4,7,10-tetraacetic acid (DOTA)-chelated Cu-64 has been explored as a PET probe, and a study showed accumulation of the probe in tumors, indicating targeting to the ligand. Neuronal stem cell-labeled SPIOs were implanted into a brain trauma patient. MR images showed the labeled cells proliferated and migrated throughout the brain tissue post-implantation [74]. The cardiomyocyte-labeled SPIOs have been used for treating plaques in atherosclerosis in which the viability of the cardiomyocytes was assessed [75]. Traztuzumab is an antibody targeting human epidermal growth factor receptor 2 (HER2) associated with breast cancer. When the HER2 antibody was functionalized into different nanoparticles of approximately 200 nm, the specific binding of the HER2 receptor to cancerous breast tissue was enhanced, and this can thus be used as a theranostic probe. The shape of the nanoparticles also impacts the targeting ability of Traztuzumab, with nanorods showing almost twice the binding affinity to HER2 receptors in a breast cancer cell line in comparison to nanospheres [76]. Antibodies targeting intracellular adhesion molecule (ICAM) and vascular cell adhesion molecule (VCAM) have been used for studying inflammation related to atherosclerosis and plaques, respectively [77,78]. Nano-biomaterials have been widely used in cell tracking and regenerative medicine. For cell labeling, the nano-biomaterials need to be inert, be easily taken up by the cells, and provide a platform for sensitive signal imaging. Thus, nano-biomaterials are usually coated with inert coating agents such as cyclodextran, followed by further functionalization with cell-penetrating peptides such as the transactivator of transcription (TAT) peptide to enhance the uptake of the nanoparticles by the cells [79].

Monoclonal antibodies used as targeting moieties for several cancer therapies have been reported [80]. Efforts have been emphasized in the research on engineering of monoclonal antibodies for the targeting. For example, antibodies can be broken down into smaller fragments that retain the receptor-binding capabilities but are easier to manipulate. The targeting efficacy generally depends on the quantity of targeting moieties and the conjugation mode. Nanomaterials that are conjugated with targeting moieties with multiple sites such as multivalency normally possess enhanced affinity, slower disassociation, and better biodistribution when compared to monovalency [81]. Some small nucleic acids such as aptamers have been attracting interest as targeting moieties. Several reports showed aptamers have a high affinity binding with various receptors, with applications in a variety of therapeutics [82,83]. Moreover, the aptamers have advantages of synthesis across a large scale and high batch-to-batch consistency, even in vitro chemical techniques [84].

Some over-expressed receptors have been chosen as targets in certain tumors for molecular imaging. Folic acid as a water-soluble vitamin has been studied as an active targeting moiety for some rodent tumor models such as ovarian cancer and prostate cancer because it binds with the Frα receptors that are over-expressed in these tumors [85]. As Frα receptors are constantly replenished on the surface of the tumors [86], folic acid has been bound to fluorescent isothiocyanate (FITC) for clinical surgeons to resect the tumors precisely [87].

## 4. Nanomaterials for Theranostic Nuclear Imaging Probes

Radiolabeled peptide or antibody can be incorporated onto nanoparticles or nanomaterials as PET (SPECT) imaging probes for cancer diagnostics as these nanoparticles demonstrated high affinity and selectivity for receptors that are over-expressed by various human cancers such as breast, lung, and prostate tumors [10,88,89]. Radiolabeled particulate nanocarriers such as ^99m^Tc-labeled colloidal nanoparticles can be used for the imaging and localization of sentinel lymph nodes [90]. In order to improve the resolution and sensitivity, ^68^Ga-labeled mannosylated human serum albumin (MSA) has been developed as a PET probe for lymph node imaging [16,91]. Peptides targeting galectin-3, beta-galactoside-binding animal lectins overexpressed by a variety of human cancers, especially breast cancer, have been successfully radiolabeled with ^68^Ga in gelatin nanoparticles (GNPs) using phage display techniques [92]. The ^68^Ga-labeled GNPs showed high radiochemical purity and stability in serum over a 4-h time. GNPs demonstrated promising results as colloidal carrier systems for drug delivery due to their biocompatibility and biodegradability. In addition, radiolabeling showed little impact on the characteristics of the GNPs. Peptide Nucleic Acid (PNA) peptide nanoparticles containing Kirsten rat sarcoma viral oncogene homolog (KRAS) mRNA and insulin-like growth factor 1 (IGF1) receptor were conjugated with DOTA-chelated ^64^Cu for tumor targeting in human pancreas cancer xenograft mice with higher tumor uptake in PET images [93].

Liposomes, especially PEGylated long-circulating liposomes, have been widely used as nanocarriers for drug delivery. Ranging from 50–1000 nm, these sphere vessels composed of lipid bilayers surrounding the aqueous core have shown great potential as theranostic nuclear imaging probes [17]. Manipulations can be applied to both the aqueous core and phospholipid bilayer, making liposomes a versatile candidate as drug carriers and imaging probes in multimodal imaging reporters and therapeutic drugs. Since liposomes and lipid nanoparticles have potential as drug carriers, evaluation of the biodistribution, pharmacokinetics, and stability in vivo is necessary, which can be achieved through nuclear imaging. Radiolabeling of lipid nanoparticles includes direct labeling of radionuclides on the surface of lipid nanoparticles and/or via chelator-based radiolabeling in which the chelator normally conjugates to the phospholipid at the surface of liposomes [94,95]. A comparison study using PEGylated liposomes/micelles with DTPA derivative was shown to be suitable for radiolabeling with various radiometals such as ^68^Ga, ^111^In, ^99m^Tc, and ^177^Lu for different imaging modalities along with specific targeting activity [96]. Solid lipid nanoparticles (SLNs) have also been employed as potential drug carriers. The in vivo evaluations were carried out using PET imaging via radiolabeled ^64^Cu through the incorporation of lipid-PEG-BAT chelator into SLNs [97]. SLNs were shown to be compatible with ^64^Cu radiolabeling, enabling biodistribution studies to be quantitatively evaluated. Through the ability to evaluate in vivo studies through nuclear imaging, potential theranostic systems using liposome drug carriers can be discovered to target more diseases.

As the structures of liposomes can be easily manipulated, radiolabeling of liposomes and lipoproteins has been explored further in the functionalization of multiple imaging reporters and/or therapeutic drugs. Natural high-density lipoprotein (HDL) nanoparticle is specific for macrophages. The reconstituted HDL was developed and radiolabeled with ^89^Zr for PET-based imaging of tumor-associated macrophages (TAM) in a breast cancer model [18]. PEG-conjugated desferroxamine B (PEG-DFB) nanocarriers were developed as a diagnostic surrogate to investigate tumor accumulation susceptibility for a cancer drug [19]. PEG-DFB nanocarriers were radiolabeled with ^89^Zr for tumor imaging using microPET/CT. Interestingly, the PEG-DFB nanocarriers exhibited passive tumor targeting properties and longer serum stability and retention time in tumors, enabling higher tumor exposure. The EPR effect of PEG-conjugated nanocarriers (15 nm) led to a long elimination half-life of the ^89^Zr-radiolabeled nanocarriers, resulting in remarkably high tumor uptake. The benzodithiophene–diketopyrrolopyrrole (BDT-DPP) conjugated polymer by Stille coupling of diketo–pyrrolopyrrole (DPP) and benzo-dithiophene (BDT) was used to synthesize biocompatible nanoparticles by self-assembly of phospholipid-PEG (DSPE-mPEG5000), followed by non-covalent binding of DOTA-chelated ^64^Cu [20]. The resulting ^64^Cu-labeled nanosystem was evaluated for its accumulation in HepG2 tumors. Both PET imaging and biodistribution studies showed high uptake in tumors 4 h post injection. The strong NIR absorption and photoacoustic imaging (PAI) sensitivity of the nanosystem indicated it can be used as dual-mode imaging probe for PET and PA. Chelator-free radiolabeling of organic lipid-based nanosystems containing PEG-lipid functionalized sphingomyelin nanometric emulsions was coupled to ^18^F-labeled fluorobenzamide-n-ethylmaleimide ([^18^F]FBEM) for in vivo biodistribution and PET imaging studies [21]. Apart from enhancing signals, sensitivity, and specificity to targets, radiolabeled nanocarriers can also be manipulated to achieve stability in plasma and long blood half-life. Radiolabeled nanosystems demonstrated high potential as targeted PET/SPECT imaging probes in cancer diagnosis Highly specific vascular targeting by radiolabeled polymeric liposomes nanocarriers for biomedical applications was also achieved through the conjugation of clickable monoclonal antibodies (mAb) and single-chain variable fragments (scFv) [98]. ^111^In radionuclide was incorporated into the click immunoliposomes, and in vivo imaging was carried out via microSPECT/CT.

Natural melanin has an inherent ability to chelate various metal ions. This property allows radiometals to be chelated on melanin, enabling the usage of nuclear medicine and imaging for theranostic applications. Water-soluble melanin nanoparticles (MNPs) have the ability to retain chelating characteristics without the need for surface modifications [22]. PEGylated melanin nanoparticles (PEG-MNPs) were radiolabeled with ^64^Cu for PET/CT imaging. With the advantage of the EPR property, ^64^Cu-PEG-MNPs, tumor accumulation was monitored, and the evaluation of ^64^Cu-PEG-MNPs as a potential targeted thernostic drug for cancer therapy was carried out. Similarly, dopamine melanin nanoparticles (DMNs) also exhibit metal-chelating properties. DMNs were used to chelate to various radiometals (^64^Cu, ^89^Zr, and ^177^Lu) for tumor PET imaging [99]. Radionuclide ^124^I was also radiolabeled to DMNs through an electrophilic substitution reaction instead of a chelation reaction. To enhance tumor targeting, folic acid was conjugated on the surface of PEG-DMNs. In order to achieve deeper tissue penetration without macrophage recognition, biocompatible melanin nanoparticles of less than 20 nm were synthesized and functionalized with PEG. To tap into the pH-induced aggregation properties, citraconic amide was coated on PEG-MNPs under mildly acidic conditions to form pH-triggered MNP aggregates that accumulated at tumor sites [100]. Through PET imaging using radiolabeled ^68^Ga radiolabeled PEG-MNPs, an enhanced tumor signal was observed in the H22 tumor-bearing mice, indicating targeted tumor imaging.

**Figure 1:**
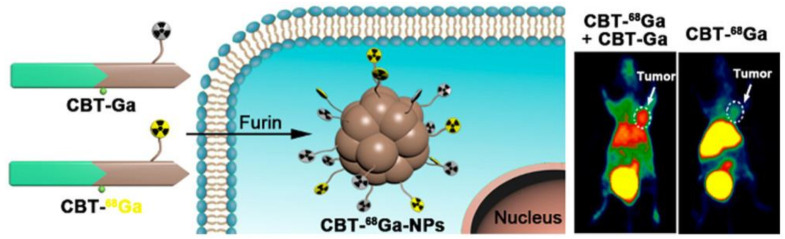
Schematic illustrations of furin-controlled condensation of CBT-^68^Ga and CBT-Ga to yield hybrid oligomers that self-assemble into radioactive nanoparticles CBT-^68^Ga-NPs in furin-overexpressing cancer cells and representative whole-body coronal microPET images of MDA-MB-468 tumor-bearing mice at 1 h post-intravenous injections of 100 µL of 5–12 MBq CBT-^68^Ga and 20 mg/kg CBT-Ga (left) or 5–12 MBq CBT-^68^Ga (right) via tail veins. Reprinted with permission from ref. [23], Copyright 2019 American Chemical Society.

Intracellular ^68^Ga-labeled nanoparticles CBT-^68^Ga were reported as a furin-responsive radiopharmaceutical for the PET imaging of furin-overexpressing cancer cells [23] (Figure 1). Incorporation of tumor-overexpressing protein convertase into intracellular nanoparticles could enhance tumor targeting and improve the cancer diagnosis. A furin-responsive Ga-labeled 2-cyano-benzothiazole (CBT-Ga) underwent furin-initiated condensation to form nanoparticles (CBT-Ga-NPs) by self-assembly. The ^68^Ga-labeled nanoparticles (CBT-^68^Ga-NPs) were used for in vivo mcroPET imaging of tumors in MDA-MB-468 breast tumor xenografts. The results showed enhanced tumor uptake in both mice, one with co-injection of CBT-^68^Ga and CBT-Ga and the other with injection of CBT-^68^Ga only. Micelles can be employed as colloidal nanocarriers for drug delivery with improved pharmacokinetics and target-to-background ratio. Micelles can be labeled with radioisotopes as a PET/SPECT imaging probe by aggregation of amphiphilic ligands comprising NOTA or DOTA coupled to an α-alkyl chain via acetate pendant arms, followed by chelation with the radionuclide [24,25]. Based on the in vivo biodistribution study, there was a directly proportional relationship between uptake of 67Ga-micelle in rat liver and the length of the pendant α-alkyl chain and lipophilicity of the amphiphilic ligand.

Inorganic nanomaterials such as mesoporous silica-based nanostructures, graphene oxide (GO), and zeolites have been explored as PET/SPECT imaging probes. Various radioisotopes such as ^64^Cu, ^68^Ga, ^89^Zr, ^18^F, and ^124^I have been reported to radiolabel silica-based nanostructures or nanoparticles through different methods including chelator-based and chelator-free labels [101]. Nano-graphene oxide (GO) sheets were modified by covalent conjugation to PEGylated chains, subsequently coupled to NOTA for radiolabeling with ^66^Ga and ^64^Cu for PET imaging in respective studies [26,27]. To enable specific targeting for CD105, a vascular target marker in tumor, the. TRC 105 antibody, was conjugated to the radiolabeled GO sheets. Accumulation of the radiolabeled-GO-TRC105 sheets was observed in tumors of 4T1 murine breast tumor-bearing mice, with steady tumor uptake over a period of time. The specificity in tumor uptake of radiolabeled-GO-TRC105 sheets indicates the potential of such nanoplatforms for targeted CD105 tumor vasculature imaging. Attachment of targeting moieties such as peptides or aptamers to zeolite Y via chloro-propyl-trimethoxysilane and azide-functionalization been reported [28]. The modified azide-functionalized zeolite nanoplatform exhibited high affinity for ^68^Ga radiolabeling for PET imaging, showing promising potential as a targeting application in imaging.

PET radioisotopes can be incorporated into quantum dots (QDs) to produce optical/PET bimodal probes. These nanomaterials with quantum confinement present unique properties for in vivo imaging studies such as biodistribution of QDs to image cancer [29,102]. Organic CdSe/ZnS QDs can be encapsulated by amphiphile polysorbate 60 for specific binding to target cells such as RGD-C18 for angiogenesis imaging. The QDs were further functionalized with 2-(p-isothiocyanatobenzyl)-NOTA ([NOTA]-C18) and then labeled with ^68^Ga for specifical accumulation in U87MG human glioma xenografts in mice by PET imaging [103]. Ultrasmall gold nanoparticles (AuNPs) have been extensively used as imaging probes such as CT, Raman or photoacoustic imaging (PAI) due to their intrinsic physicochemical properties. AuNPs with homogeneous size have been manipulated into desired shapes or tailored to be tumor-specific for theranostic applications. Radiolabeling of AuNPs with ^64^Cu (^64^Cu-AuNPs) was evaluated as a diagnostic PET imaging probe based on favorable in vivo biodistribution and clearance of ^64^Cu-AuNPs [104]. The ^64^Cu-labeled palladium-gold core-shell tripod nanomaterial ^64^Cu-doped PdCu@Au was synthesized with ^64^Cu directly doped into the crystal lattice and functionalized with D-Ala1-peptide T-amide (DAPTA) for CCR5 breast tumor PET imaging in 4T1 mice. Due to the bimodal characteristics of ^64^Cu-doped PdCu@Au, the imaged-guided photothermal cancer treatment was promising [105]. Copper sulfide-ferritin nanocage CuS-Fn NCs were prepared via a novel biomimetic method for incorporation of ultrasmall CuS nanoparticles within the cavity of ferritin nanocages (Fn NCs). ^64^Cu-labeled ^64^CuS-Fn NCs demonstrated considerable tumor uptake in human glioblastoma U87MG-bearing nude mice in PET imaging [106]. CuS-Fn NCs have potential in cancer photothermal therapies. Cornell dots (“C dots”) were the first ultrasmall inorganic hybrid nanoparticles for clinical trials. Functionalization of C-dots with a ^124^I containing peptide (^124^I-cRGDY-PEG-C) could be used as hybrid imaging probe for translational and clinical cancer diagnostics [107]. PAI demonstrates the advantages of both of high sensitivity of optical imaging and high resolution of ultrasonic imaging. Besides that, it can be used for cancer treatment by photothermal therapy. Molybdenum-based poly oxometalates were used for redox-activated PAI-guided photothermal therapy. The PAI imaging probe was labeled with ^89^Zr to investigate its biodistribution study by PET [108].

**Figure 2:**
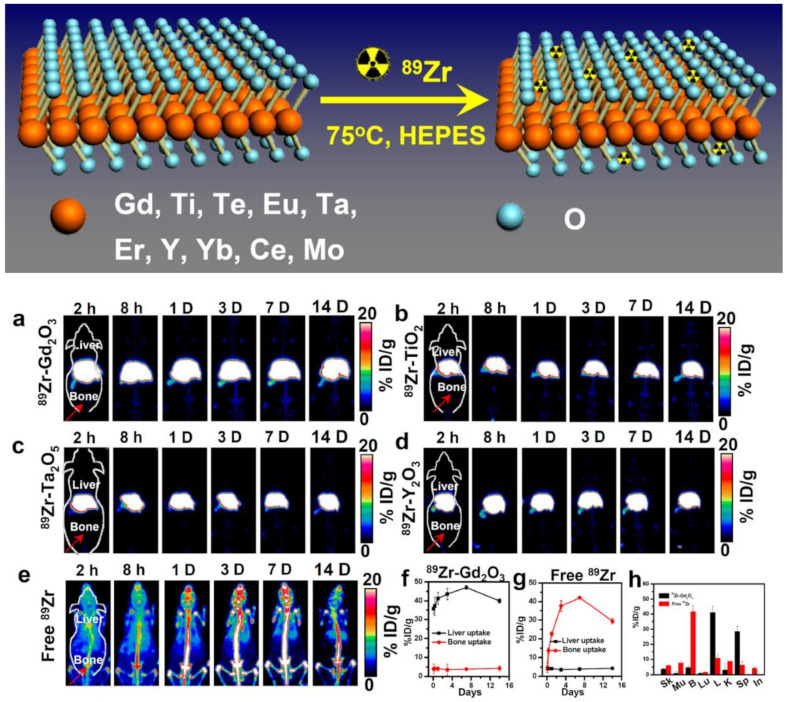
(**top**) Schematic illustration showing the chelator-free labeling of different types of metal oxides (M_x_O_y_) with ^89^Zr. (**bottom**) In vivo radiostability study using PET imaging. (**a**–**e**) In vivo maximum intensity projections (MIPs) of mice after i.v. injection of ^89^Zr-M_x_O_y_-PEG nanomaterials: (**a**) ^89^Zr-Gd_2_O_3_-PEG; (**b**) ^89^Zr-TiO_2_-PEG; (**c**) ^89^Zr-Ta_2_O_5_-PEG; (**d**) ^89^Zr-Y_2_O_3_-PEG), and (**e**) Free ^89^Zr at different time points. (**f**,**g**) Quantitative region of interest (ROI) analysis of the dynamic uptake of ^89^Zr after i.v. injection of ^89^Zr-Gd_2_O_3_-PEG (**f**) or free ^89^Zr (**g**) in bone and liver. (**h**) Biodistribution of ^89^Zr-Gd_2_O_3_-PEG and free ^89^Zr measured at 14 days p.i. Data are presented as the percentage of injected dose per gram of tissue (%ID/g): Sk, skin; Mu, muscle; B, bone; Lu, lung; L, liver; K, kidney; Sp, spleen; In, intestine. Error bars are based on the standard error of the mean (SEM) of triplicate samples. Reprinted with permission from ref. [30], Copyright 2017 American Chemical Society.

MRI/PET multimodal nanomaterial probes are promising for tumor imaging [109,110,111]. There two types of MRI/PET multimodal nanomaterial probes are based on classifications of MRI contrast agents: magnetic nanoparticles such as superparamagnetic iron oxide [112,113] and gadolinium containing nanomaterials [114]. Chelator-free radiolabeling of ^89^Zr to a variety of metal oxide (M_x_O_y_, x = 1–2, y = 2–5) nanomaterials with a PEGylated surface were reported as PET/MR probes for in vivo lymph node (LN) mapping [30] (Figure 2). The high-labeling yield M_x_O_y_ nanomaterials showed stability in serum due to the strong bonding between oxyphilic ^89^Zr^4+^ with the oxygen atom on the M_x_O_y_ surface. The nanostructures or different morphologies of metal oxides affect the target-ligand affinity.

A dendrimer-based single molecular platform for merging MRI and PET probe moieties to facilitate colocation and cross-validation from each modality in targeted regions of interest was reported [31]. The platform contained NOTA for specific chelation with ^67/68^Ga and DOTA for Gd. The platform also carried three amine groups to attach targeting moieties for enhanced targeting [32]. Conjugation of the ^64^Cu-DOTA complex with dextran-coated iron oxide nanoparticles was specially modified with maleic anhydride to obtain a high negative surface charge. The increased anionic charge enhanced the scavenger receptor type A (SR-A) targeting in which SR-A is expressed in macrophages. Polymeric micelle nanoparticles were modified with Fe-deferoxamine (Fe-DFO) as an MR contrast agent to avoid Gd-related toxicity such as nephrogenic systemic fibrosis and were labeled with ^89^Zr as a dual modality probe for tumor imaging through EPR effects [33]. Human serum albumin (HSA)-encapsulated IONPs were labeled with ^64^Cu-DOTA and NIR dye Cy5.5 as an optical/PET/MRI triple modal nanoprobe [34]. Prolonging the NPs’ blood circulation of the HSA matrices led to significant accumulation of NPs in the lesion of a U87MG (glioblastoma cell)-xenografted mouse model by in vivo trimodal imaging. Thermally cross-linked IONPs as a triple modal optical/microPET/MRI probe were synthesized by a facile preparation method through single labeling with positron-emitting ^124^I [35]. The β^+^decays of ^124^I with a mean energy led to strong Cerenkov radiation for Cerenkov luminescence imaging.

Radioiodination of bio-nanomaterials can be easily carried out due to the abundance of tyrosine and histidine amino acid groups inside liposomes, polymers, antibodies, peptides, and extracellular vesicles [10]. Similarly, methods of radiolabeling of nanomaterials with iodine are through encapsulation and/or surface attachment depending on the type of nanomaterial and its applications. By combining the uses of nanomaterials as drug carriers and nuclear imaging, iodine-containing radiopharmaceuticals can be developed as therapeutic and diagnostic medicines for various diseases [101,115,116]. ^131^I was radiolabeled on a polyethylenimine (PEI)/doxorubicin (DOX) complex, forming radiolabeled nanoparticles with pH-response for cellular uptake by conjugating alkoxyphenyl acylsulfonamide (APAS) functional groups to the surface of the complex. APAS in the radiolabeled APAS-^131^I-PNPs/DOX nanoparticles changed their surface charges from neutral charge at physiological pH (pH ~7.4) to positive charge at slightly acidic pH (pH ~5.5–6.0), which allows cellular uptake of nanoparticles through electrostatic interaction tumor cell membranes. In vitro SPECT imaging showed enhanced signal of C6 cancer cells with APAS-^131^I-PNPs/DOX compared to that of cells with ^131^I-PNPs/DOX at tumor pH, while at physiological pH ~7.4, lowered signals were found in both cells. Similarly, in vivo SPECT imaging of xenograft tumors showed enhanced signal at 4-, 6-, 8-, and 12-h post-injection in tumor-bearing mice injected with APAS-^131^I-PNPs/DOX compared to low signals of tumor-bearing mice injected with ^131^I-PNPs/DOX throughout the same time intervals. The results suggest that APAS promotes cellular uptake of the nanoparticles under slightly acidic conditions (i.e., tumor pH) [36].

Shikonin, a napthoquinone often used in traditional Chinese medicine for wound healing, is able to suppress tumor cell growth and induce apoptosis of cancer cells [117,118]. The synergistic effect of shikonin and silver nanoparticles (AgNPs) was investigated for theranostics in lung cancer. Shikonin was not only used as anti-tumor treatment for A549 cells (human lung carcinoma cell line) but also acted as a reducing and stabilizing agent for the synthesis of spherical shikonin-AgNPs, enabling a more environmentally friendly synthesis reaction. Through radioiodination of ^131^I to the synthesized shikonin-AgNPs, biodistribution studies by gamma counting showed their highest preferential retention time in the lung tissues at all-time points compared to other organs, indicating specific targeting in lungs. Moreover, shikonin-AgNPs also showed inhibitory effects for A549 cells in cell viability and proliferation [37]. AuNPs were used as delivery cargos to deliver boron atoms to cancer cells for boron neutron capture therapy (BNCT). PEGylated AuNPs were under surface modified to conjugate an anti-HER2 antibody 61 IgG for specific tumor targeting. The resulting AuNP-boron cage assemblies (B-AuNPs) were radiolabeled with ^123^I as a SPECT imaging probe for N87 gastric cancer xenograft model targeting [38]. Both biodistribution and microSPECT/CT imaging studies showed high uptake of ^123^I-61-B-AuNPs in tumors, indicating the probe’s promising theranostic applications for BNCT in clinical settings. HSA has a target-specific binding ability for glycoprotein60, which is commonly found on the surface of cancer cells. HSA containing nanoparticles thus can be used as a site-specific drug cargo to deliver drugs to targeted sites [119]. HSA containing metabolizable nanomaterial was reported to track tumor variation under X-ray exposure. The SPECT/CT imaging demonstrated prolonged retention time of radiolabeled ^125^I-HSA nanoparticles in the tumor due to X-ray exposure compared with the control tumor imaging without X-ray effects [39].

## 5. Radiolabeled Exosomes for Nuclear Imaging

Exosomes were first discovered in the 1980s when it was hypothesized that the debris of these nanosized vesicles was secreted into the extracellular space from cells during blood reticulocyte maturation to remove unwanted transferrin receptors [120,121,122,123,124,125]. Until 1996, these exosomes were considered debris, but since then, studies have reported the role of exosomes in immune responses such as antigen presentation in immune-modulating activity of B cell-derived vesicles and their potential in immunotherapy [123,126,127,128]. Extensive research has been done in recent years to gain a deeper understanding of these complex vesicles and their involvement in the cellular system, as well as exploration for potential clinical diagnosis and therapeutics in nanomedicine [125,129,130]. Currently, there are studies to investigate the effects of exosomes on SARS-CoV-2 coronavirus and potential development of exosome-based therapeutic approaches to tackle this deadly virus [131,132,133]. Evidently, exosomes play a crucial role not only in elimination of unwanted proteins out of the cell but also in other important cellular communications and functions [122,123,126,127,129,134,135,136].

There are three main subgroups of extracellular vesicles (EVs) that are classified based on their biogenesis origin: (a) exosome (30–100 nm), (b) microvesicles (100 nm–1 μm), and (c) apoptotic bodies (1–5 μm). In this review, only exosomes will be explained as the focus is on nanosized materials. Exosomes are a subgroup of EVs secreted as nanosized particulates into the extracellular space when multivesicular bodies fuse with the cell membrane. Many types of cells found throughout the body produce exosomes including mesenchymal stromal cells (MSCs), nervous system cells (Schwann cells), neurons, epithelial cells, dendritic cells, fibroblasts, and cells of the immune system such as T-cells, B-cells, and macrophages. Furthermore, these nanoparticulate vesicles can be found in bodily fluids such as blood, breast milk, urine, sperm, amniotic fluid, saliva, cerebrospinal fluid, and many more [122,124,127,128]. Exosomes play a vital role in intercellular communication, acting as a “transporter” in carrying bioactive materials such as proteins, lipids, RNAs, and DNAs that will be taken up by target cells to elicit signals important for regulation, regeneration, and protection in the body systems. Additionally, the involvement of exosomes in cancer progression has also been evidently reported; tumor-derived exosomes (TDEs) are known to be involved in cancer progression [124,125,126,130]. One of the greatest advantages of these exosomes is the ability to cross biological barriers such as the blood–brain barrier easily, and therefore, bypassing potential side effects arising from traditional cell-based therapy [40,125,129,137]. For example, pulmonary embolism that could potentially arise from MSC transplantation in the brain could be avoided [132,133]. Making use of the advantageous properties of exosomes may provide endless potential applications in both clinical diagnostic and therapeutic tools.

Since exosomes encapsulate many bioactive materials important for paracrine factors, tumor growth, and suppression, these vesicles can serve as biomarkers for diagnosis and prognosis for diseases [124,128,129,138]. Studies have shown that exosome concentrations were found to be higher in the blood of patients with cancer [124,127,128]. Additionally, radiolabeled exosomes can also be used for tracking of pharmacokinetics via different routes of administration, which is important in biodistribution studies in determining the design of the drug [40,138,139] for clinical translation.

The radioisotope can be labeled through two main techniques: (a) surface labeling and (b) intraluminal labeling. The surface labeling methods for exosomes that have been investigated so far include modifications of genetic materials, direct labeling of radioisotopes, and attachment with the help of a bifunctional chelator. Intraluminal radiolabeling encapsulates the radioisotope with the help of a radiometal complex into the intravesicular space of the exosomes. There are two methods currently known, remote loading and ionophore-chelator binding. Further elaborated details for each labeling method can be found [129,140]. The versatility of exosomes provides a platform for new discoveries in theranositcs. Even so, a current challenge faced by scientists in labeling exosomes is maintaining the integrity of the exosomes. Any type of modification, for example, surface labeling, could affect the properties of the exosome, which can nullify the potential use of the radiolabeled exosome as a diagnostic or therapeutic tool in clinical application [130,138,139]. In addition, the exact location where the radionuclide binds in the exosome is still uncertain which may compromise imaging [129]. Nonetheless, it is worth investigating methods in modifying exosomes to improve the uptake or physiochemical properties, creating novel techniques for diagnosis and therapeutics. By labeling a radionuclide on an exosome, nuclear imaging such as SPECT and PET can be utilized, which is particularly useful in acquisition of whole-body imaging in clinical translation. Despite the superior sensitivity and deep tissue penetration capacities, there are only few studies reporting the use of radiolabeled exosomes [40,140,141].

^131^I-exosomes are potentially used for disease prognosis, exosome-based therapies, and monitoring binding ability to target tissues via SPECT/CT. In vivo tumor model mice imaging showed higher tumor uptake of ^131^I-tumor-derived exosomes (^131^I-TDE) compared to the tumor uptake of free ^131^I in the same tumor model [141]. The biodistribution studies showed unlike ^131^I-HEK293 exosomes (human embryonic kidney 293 cells that are non-cancerous), ^131^I-MDSCs exosomes (myeloid derived suppressor cells) and ^131^I-EPCs exosomes (endothelial progenitor cells) accumulated in the primary tumor site in breast cancer and metastatic site in lung cancer, indicating target-specific TDEs accumulation in tumors, enabling the potential uses of the specific derived exosomes.

**Figure 3:**
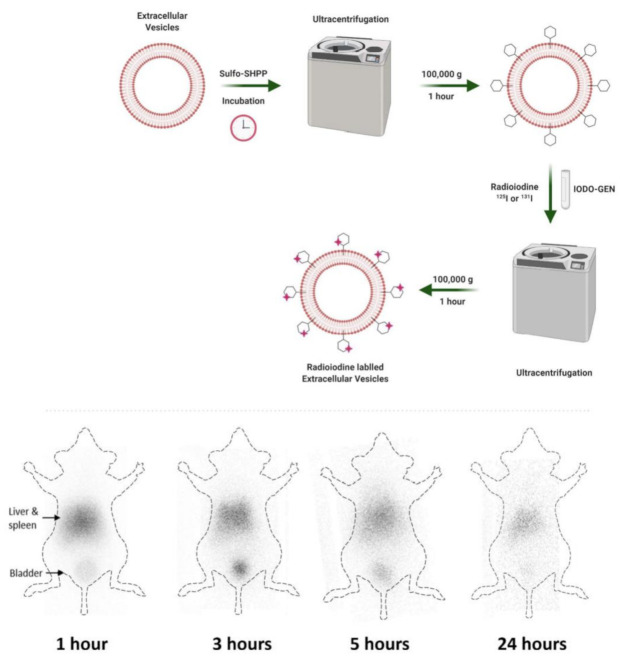
(**top**) Schematic of radioiodine labeling of extracellular vesicles. (**bottom**) In vivo imaging of I-131-Cal62-EVs. After intravenous injection of I-131-Cal-62-EVs (3.7 GBq), gamma camera images were acquired at 1 h, 3 h, 5 h, and 24 h in BALB/c nude mice. The gamma camera images showed intense uptake in liver and spleen areas. There was intense trace accumulation in the bladder. Reprinted from ref. [41].

A novel radiolabeling approach was reported for ^131^I-labeled EVs for two different cell lines, thyroid cancer cell (Cal62) and natural killer cells (NK92-MI). Instead of direct labeling the radioiodine on the EV surfaces, sulfosuccinimidyl-3-(4-hydroxypheynyl) propionate (SULFO-SHPP) was employed to modify the surface of EVs to increase the number of available sites for radioiodination on the EV surfaces. In terms of morphology and size, the SEM images showed little differences between normal EVs in comparison to ^131^I-labeled surface modified EVs. A gamma camera was initially used for in vivo tracking to quantify the levels of free and radiolabeled exosomes. Due to the low resolution of gamma camera, it was unable to perform imaging of the thyroid gland, but limitations can be overcome by using SPECT/PET imaging [41] (Figure 3). Through modification of the surface of EVs, increased labeling sites can be labeled with radioiodine for in vivo studies and eventually clinical potentials. Similarly, the glycosylated moieties on EV surfaces were modified followed by conjugation of ^124^I- for in vivo studies using different routes of administration. By PET-CT imaging and biodistribution studies, the radioactivity in different organs was quantified at different time intervals with a gamma counter. The glycosylated EV surface affected the biodistribution of ^124^I-EVs in different organs after 72 h. Glycosylated ^124^I-EVs by neuraminidase showed higher accumulation of radioactivity in the lungs than unmodified ^124^I-EVs. Interestingly, the presence of glycosylated ^124^I-EVs was detected in the brain. This offers an example of how surface modification of EVs for radiolabeling could affect the distribution and how different routes of administration could lead to different biodistributions of the radiolabeled EVs [40].

^64^Cu- and ^68^Ga- radionuclides chelated by a bifunctional chelator, 1,4,7-triazacyclononane-triacetic acid (NOTA), were used to label exosomes via the conjugation of NOTA to amine groups on the exosome surfaces. ^64^Cu- or ^68^Ga-labeled exosomes showed similar accumulation results at the target sites in PET imaging. Due to the difference of half-life and labeling efficiency of the two radioisotopes, ^64^Cu- was superior, with a half-life of 12.7 h and a labeling efficiency of 13.3% for 100 ug of exosomes, compared to ^68^Ga-, with a half-life of 68 min and a labeling efficiency of 2.22% for 100 μg exosomes. The study also highlighted the superiority of PET imaging having better sensitivity and depth penetration over optical imaging where accumulation of radiolabeled exosomes was found in the lungs and liver using PET imaging but was not found in optical imaging. The radiolabeling of exosomes using bifunctional chelators such as NOTA enables less dependency on the cell type since most cells contain amine functional groups for interaction [42]. With less dependency on cell type, more exosomes derived from different tumors and cells could be used as nanomaterials for radiolabeling in nuclear medicine and imaging.

Intraluminal labeling of radionuclides to exosomes was demonstrated through the use of a metastable lipophilic [^89^Zr]Zr(oxinate)_4_ neutral complex. The radiometal complex was encapsulated into the exosomes and ensured that the surfaces of the exosome were not affected. The lipophilic complex [^89^Zr]Zr(oxinate)_4_ was able to pass through the lipid bilayer of the vesicles where ^89^Zr dissociated from the oxine ligands; dissociated free ^89^Zr then bound to intravesicular metal-chelating ligands, such as proteins and nucleic acids within the exosomes. Biodistribution studies using exosomes derived from pancreatic cancer cells (PANC1) via PET-CT displayed ^89^Zr-PANC1 exosomes accumulated in the liver, spleen, bladder, lymph nodes, and brain within 1 h of intravenous (iv.) injection [43]. As exosomes are involved in cellular communication, the integrity of the surface of exosomes is crucial. Modifications could result in impacts on the distribution and functions of the exosomes. This work has shown a radiolabeling method that could prevent alterations of the surface of exosomes, expanding routes to radiolabel exosomes. The possibilities of discovering new probes to label these nanosized vesicles could potentially help in creating novel clinical diagnostic and therapeutic tools in nuclear medicine to tackle different targets and diseases.

## 6. Conclusions

With the development of nuclear imaging probes using nanomaterials, pathological processes at molecular levels can be revealed that could potentially help in early detection and interventions of various diseases. Enhancement of nanomaterial targeting capabilities as imaging probes has been explored through various approaches such as surface modifications and targeting moieties attachment. In this review, we summarized developments of nanomaterials as nuclear imaging probes through various modifications. The applications of nanomaterials have been extensively exploited for biocompatibility, in vitro stability, and in vivo biodistribution and animal imaging. Despite promising results from in vivo studies, there are still challenges that need to be overcome before nanomaterials can be applied as diagnostic and/or therapeutic nuclear medicine and imaging tools in clinical applications. For example, the reproducibility of various nanomaterials needs to be systematically determined, and the synthetic methods need to be standardized to ensure their biocompatibility so that theoretical modelling of nanomaterial integration can be guided for cell tracking and cell internalization. The review provides some future research directions to those with expertise in radiochemistry, nuclear imaging engineering, and other bio-imaging research. It is envisaged that the research thrust area combining these nanomaterials including exosomes can lead to a new generation of nuclear imaging probes and introducing a paradigm shift in theranostics.

## Figures and Tables

**Table 1:** Nanomaterial nuclear imaging probes, their characteristics, applications, and research outcomes.

Nanomaterial Probe	Isotope	Morphology, Coating, Modifications, Chelator, and Hydrodynamic Diameter, etc.	Applications and Research Outcomes	Ref.
^68^Ga-NOTA-MSA (human)	^68^Ga	NPs, SCN-mannose modified HSA, NOTA	Diagnostic PET imaging for SLN	[16]
[^64^Cu]DO3A-*KRAS* PNA-peptide	^64^Cu	NPs, DO3A	Diagnostic PET imaging, specific genetic characteristics of radiolabeled-PNA-peptide NPs	[17]
HDL	^89^Zr	NPs, PL or apoA-I conjugated, 8.6 ± 1.3 nm	Diagnostic PET imaging, ^89^Zr-labeled TAM imaging using HDL, specific for macrophages, quantitative macrophage PET	[18]
DFB	^89^Zr	Nanocarriers, PEGylated, ~15 nm	Diagnostic PET imaging, ~15 nm PEG_40kDa_-^89^Zr-radiolabeled surrogates of PEG-prodrugs of SN-38 (PLX038)	[19]
BPT-DPP	^64^Cu	NPs, spherical, PEGlyated, NOTA, 31.3 ± 2.8 mm	PET/PAI dual modality imaging probe	[20]
SNs and SNs-RPM	^18^F	Nanometric emulsions, [^18^F]FBEM conjugated, PEGylated, ~130–150 nm	Diagnostic PET imaging, ^18^F-radiolabeled technique for lipid-based nanocarriers	[21]
MNPs	^64^Cu	NPs, PEGylated, ~11 nm	Diagnostic PET imaging, good imaging, therapeutic effects on A431 tumors, potential in targeted radiotherapy	[22]
CBT-^68^Ga-NPs	^68^Ga	NPs, 258.3 ± 127.85 nm	Diagnostic PET imaging, tumor-targeted imaging probe	[23]
Micelles	^67^Ga	Nanocarriers, α-alkyl chain, NOTA or DOTA	Diagnostic PET imaging, higher uptake in liver of micelles due to increased lipophilicity of Ga(III) chelates	[24,25]
Nano-GO sheets	^66^Ga ^64^Cu	PEGlylated, NOTA, 10–50 nm	Diagnostic PET imaging, site-specific tumor neovasculature targeting through functionalization of the TRC105 antibody	[26,27]
Zeolite Y	^68^Ga	Nanocrystalline, azide functionalized, µm to ~55 nm	Diagnostic PET imaging, incorporation of ^68^Ga- to pores of azide-functionalized NaY zeolite as a bifunctional molecular targeting vector	[28]
QDs	^64^Cu	Amine functionalized, DOTA, ~20–25 nm	PET/NIRF, dual modality imaging QD-based nanoprobe for tumor VEGFR expression	[29]
Metal oxides	^89^Zr	Nanorods, nanospheres, NPs, PEGylated, ~140 nm	Diagnostic PET/MRI imaging, chelator-free radiolabeling of ^89^Zr- on metal oxide for multimodal imaging	[30]
Dendrimer-based single molecular platform	^67/68^Ga	NPs, NOTA and DOTA	PET/MRI or SPECT/MRI dual modality imaging probe with quantifiable radioisotopes chelated in NOTA or DOTA	[31]
MDIO-^64^Cu-DOTA	^64^Cu	IONPs, dextran coated, DOTA, ~62.7 nm	PET/MRI dual-modality imaging probe, Anionic charges on surface of nanoparticulate MDIO-^64^Cu-DOTA to facilitate recognition by SR-A on macrophages for VAP	[32]
^89^Zr/Fe-DFO micelles	^89^Zr	Micelles, ~25–50 nm	PET/MRI dual modality imaging probe, with Fe-DFO for MRI, high tumor-to-blood and tumor-to-muscle ratio, on EPR-based tumor imaging	[33]
HAS-IONPs	^64^Cu	Dopamine and Cy5.5 coating, DOTA, ~29.4 ± 1.2 nm	PET/NIRF/MRI triple-modality imaging probe, dopamine and Cy5.5coating IONPs encapsulated in HSA matrices	[34]
TCL-SPIOs	^124^I	IONPs, PEGylated, tyramine coating, 39 ± 8 nm	Optical/PET/MRI triple-modality imaging probe through adaptation of Cerenkov radiation	[35]
APAS-^131^I-PNPs/DOX	^131^I	Nanoparticulate platform, PEGylated, 241.16 ± 13.57 nm	Theranostic (SPECT, chemotherapy), enhanced cellular uptake in cancer cells by smart theranostic system, enhanced SPECT imaging and chemo/radioactive combination cancer therapy	[36]
Shikonin-AgNPs	^131^I	NPs, spherical, modified by shikonin, ~106 nm	Theranostic (Gamma-counter, therapy), cell viability and proliferation of human lung carcinoma cell inhibited by synergistic antitumor combinatorial therapy	[37]
^123^I-61-B-AuNPs	^123^I	NPs, PEGylated, ~54.48 ± 14.72 nm	Theranostic (SPECT/CT, BNCT) HER2-targeting boron-containing AuNPs for specific tumor localization and tracking, antibody modified boron containing AuNPs for BNCT	[38]
^125^I-HSA	^125^I	Nanodrugs, Cy5.5	Theranostics (SPECT/CT, radiation-based therapy), enhanced cell uptake under X-ray exposure, prolonged tumor retention time, positive correlation between cell uptake and Caveolin-1 expression, albumin-based combination therapy	[39]
Exosomes/Extracellular Vesicles (EV)	^124^I	Nanovesicles, neuraminidase modified, ~100 nm	Diagnostic PET imaging, tracking quantitatively of radiolabeled EVs with neuraminidase modification on EV surface	[40]
	^131^I/^125^I	Nanovesicles, SULFO-SHPP conjugated, 233.8 ± 32.7 nm)	PET imaging, surface modification of EVs with linker SULFO-SHPP and radioiodine labeling on linker	[41]
	^64^Cu/^68^Ga	Nanovesicles, Cy7, NOTA, ~100 nm	Optical/PET dual imaging probe, less dependency on cell type for exosome biodistribution in mice	[42]
	^89^Zr	Nanovesicles, <~150 nm	Diagnostic imaging (PET), direct radiolabeling of [^89^Zr]Zr(oxinate)_4_ to target internal components of EVs without surface modifications	[43]

## Abbreviations

3D: three-dimensional; [^18^F]FBEM: ^18^F-labeled fluorobenzamide-n-ethylmaleimide; ^131^I-TDE:^131^I-tumor-derived exosomes; AgNPs: Silver nanoparticles; AuNPs: Gold nanoparticles; APAS: Alkoxyphenyl acylsulfonamide; BBB: blood–brain barrier; BDT-DPP: Benzodithiophene-diketopyrrolopyrrole; BNCT: boron neutron capture therapy; C dots: Cornell dots; CNS: Central Nervous System; CT: Computed tomography; cRGD: cyclic arginylglycylaspartic acid; DAPTA: D-Ala1-peptide T-amide; DFB: desferroxamine B; DMNs: Dopamine melanin nanoparticles; DOTA: 1,4,7,10-tetraazacyclododecane-1,4,7,10-tetraacetic acid; DOX: doxorubicin; EPR: Enhanced permeation and retention; EVs: Extracellular vesicles; Fab: Antigen-binding fragment; Fe-DFO: Fe-deferoxamine; FITC: Fluorescent Isothiocynate; Fn NCs: Ferritin nanocages; GNPs: Gelatin nanoparticles; GO: Graphene oxide; HDL: High-density lipoprotein; HER2: Human epidermal growth factor receptor 2; HSA: Human serum albumin; ICAM: intracellular adhesion molecule; IGF1: Insulin-like growth factor 1; IONPs: Iron oxide nanoparticles; ^K^D: Dissociation constant; KRAS: Kirsten rat sarcoma viral oncogene homolog; mAb: Monoclonal antibodies; MDIO: maleylated dextran-coated IONPs; MNP: Melanin nanoparticles; MRI: Magnetic Resonance Imaging; MSA: Mannosylated human serum albumin; MSCs: Mesenchymal stromal cells; NIRF: near-infrared fluoroscopy; NK92-MI: Natural killer cells; nM: Nanomolar; NOTA: 1,4,7-Triazacyclononane-1,4,7-triacetic acid; QDs: Quantum dots; PEI: polyethylenimine; PEG: polyethylene glycol; PEO: polyethylene oxide; PET: Positron emission tomography; PNA: Peptide nucleic acid; PNP: multifunctional PEI; PPO: polypropylene oxide; PVA: polyvinyl alcohol; PVP: pyrrolidone; QD: Quantum dots; RES: Reticuloendothelial; RPM: CPIEDRPMC peptide; SLNs: Solid lipid nanoparticles; SNs: sphingomyelin nanometric emulsions; SPECT: Single-photon emission computed tomography; scFv: Single chain variable fragments; SR-A: Scavenger receptor type A; SSTRs: somatostatin receptors; SULFO-SHPP: Sulfosuccinimidyl-3-(4-hydroxypheynyl) propionate; TAM: Tumor-associated macrophages; TDEs: Tumor-derived exosomes; TAT: Trans-activator of transcription; TCL-SPIOs: thermally cross-linked, superparamagnetic iron oxide nanoparticles; US: Ultrasound; VAP: vulnerable altherosclerotic plaques; VCAM: vascular cell adhesion molecule.
